# Surgical management and postoperative functional recovery of distal ulna tumors: a case report and literature review

**DOI:** 10.3389/fonc.2026.1608661

**Published:** 2026-02-03

**Authors:** Yue-hua Han, Tian-jun Liang, Chen-xu Zhu, Sheng-jie Kang, Dong-lai Wang

**Affiliations:** Department of Orthopedics, The Fourth Hospital of Hebei Medical University, Shijiazhuang, Hebei, China

**Keywords:** bone metastasis, clear cell renal cell carcinoma, distal ulnar resection, malignant tumor, wrist joint function

## Abstract

**Introduction:**

Bone is the second most common site of metastasis for renal malignancies after the lung, with approximately 30% of metastatic renal cell carcinomas involving bone. Current research indicates that the common sites of bone metastasis from renal malignancies are mainly the axial skeleton. About 71% of patients with bone metastasis have multiple bone metastases. Cases of distal ulna metastasis of renal malignancies are rarely reported. At present, there are no standardized guidelines for the surgical treatment of distal ulna metastasis of renal malignancies. We report a case of distal ulna metastasis from renal malignancy. Additionally, we review the surgical techniques of distal ulna resection and their impact on wrist function over the past 20 years.

**Case Presentation:**

A 61-year-old male patient came to the hospital due to pain and swelling in the left forearm for one month. The patient had undergone surgery for clear cell renal cell carcinoma of the left kidney, and biopsy confirmed metastatic clear cell renal cell carcinoma. He required surgical resection. In this case, the patient underwent wide segmental resection of a 10 cm tumor in the distal ulna without soft tissue reconstruction. Eleven months after surgery, the patient was able to achieve complete wrist joint mobility following active rehabilitation. The patient was discharged without complications and is now undergoing regular follow-ups every three months, as well as receiving monthly doses of the bone protective agent denosumab for the treatment of bone metastasis.

**Conclusion:**

In this case, the distal ulna tumor segment was extensively resected without reconstruction or prosthetic implantation. The patient had good wrist joint function in the short term. Different surgical methods for distal ulna resection impact the recovery of wrist joint function in patients. However, as all the literature reviews involved short- to medium-term follow-ups, longer-term follow-up may be needed to observe the recovery of wrist joint function at different time points in patients.

## Background

Bone is the second most common site of metastasis for renal malignancies after the lung, with approximately 30% of metastatic renal malignancies involving bone metastasis (1). Current research indicates that the common sites of bone metastasis from renal malignancies are mainly the axial skeleton and about 71% of patients with bone metastasis have multiple bone metastases. Cases of distal extremity metastasis of renal malignancies are rarely reported. Although the mechanism of distal extremity metastasis is not yet fully understood, it is generally believed that bone metastasis, including metastasis to the distal extremity region, occurs through the bloodstream (2). Because cancer cells in the peripheral bones of the human body are diffusely distributed, it is more difficult for them to adapt, proliferate, and metastasize. Compared with the axial skeleton, the peripheral bones have relatively less red bone marrow, and their location is closer to the skin surface with lower temperature, making it more difficult for cancer cells to survive and proliferate. Because a large number of cancer cells need to disseminate for the tumor to metastasize to the distal extremities, patients with distal extremity metastasis generally have a poorer prognosis (3). Although the diagnosis and treatment models for renal malignancies are improving and the survival time of patients is gradually increasing, the risk of bone metastasis and skeletal-related events (SREs) is also increasing. SREs not only reduce the quality of life of patients, but also decrease their treatment compliance and shorten their survival time (1). Currently, there are no standardized guidelines for the surgical treatment of distal extremity bone metastasis. We report a case of distal ulnar metastasis from renal malignancy and review various surgical methods for distal ulnar resection and the recovery of wrist joint function in patients.

## Case report

**Figure 1:**
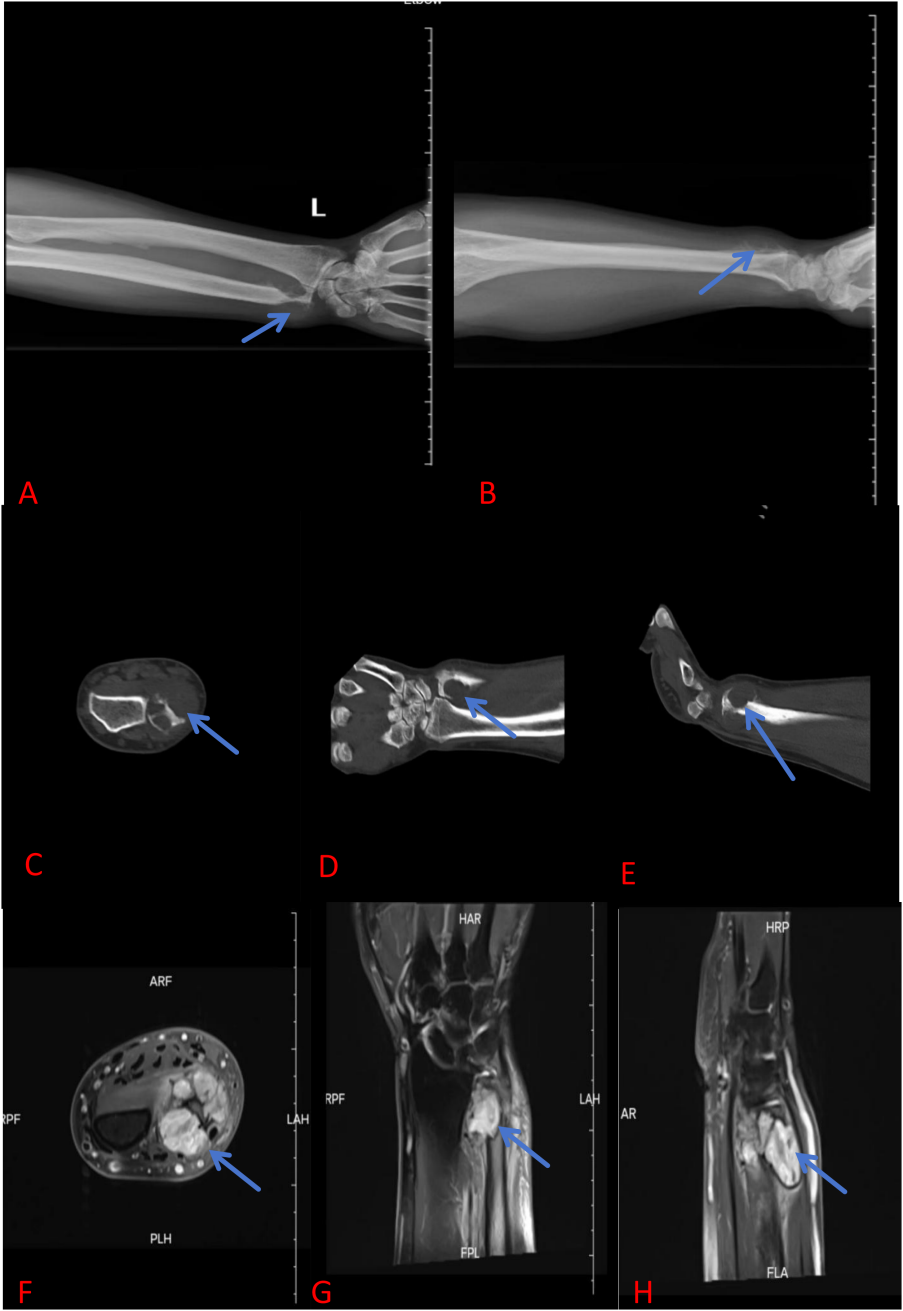
**(A)** Latera X-ray of the left ulna. **(B)** Anteroposterior X-ray of the left ulna: Expansive tumor at the distal ulna. Left ulna CT: There is obvious bone destruction at the distal ulna. **(C)** Transverse position. **(D)** Coronal position. **(E)** sagittal position. Left ulna MRI: a soft tissue mass at the distal end of the left ulna, measuring 3.9cm × 3.7cm × 3.5cm. **(F)** Transverse position. **(G)** Coronal position. **(H)** sagittal position.

**Figure 2:**
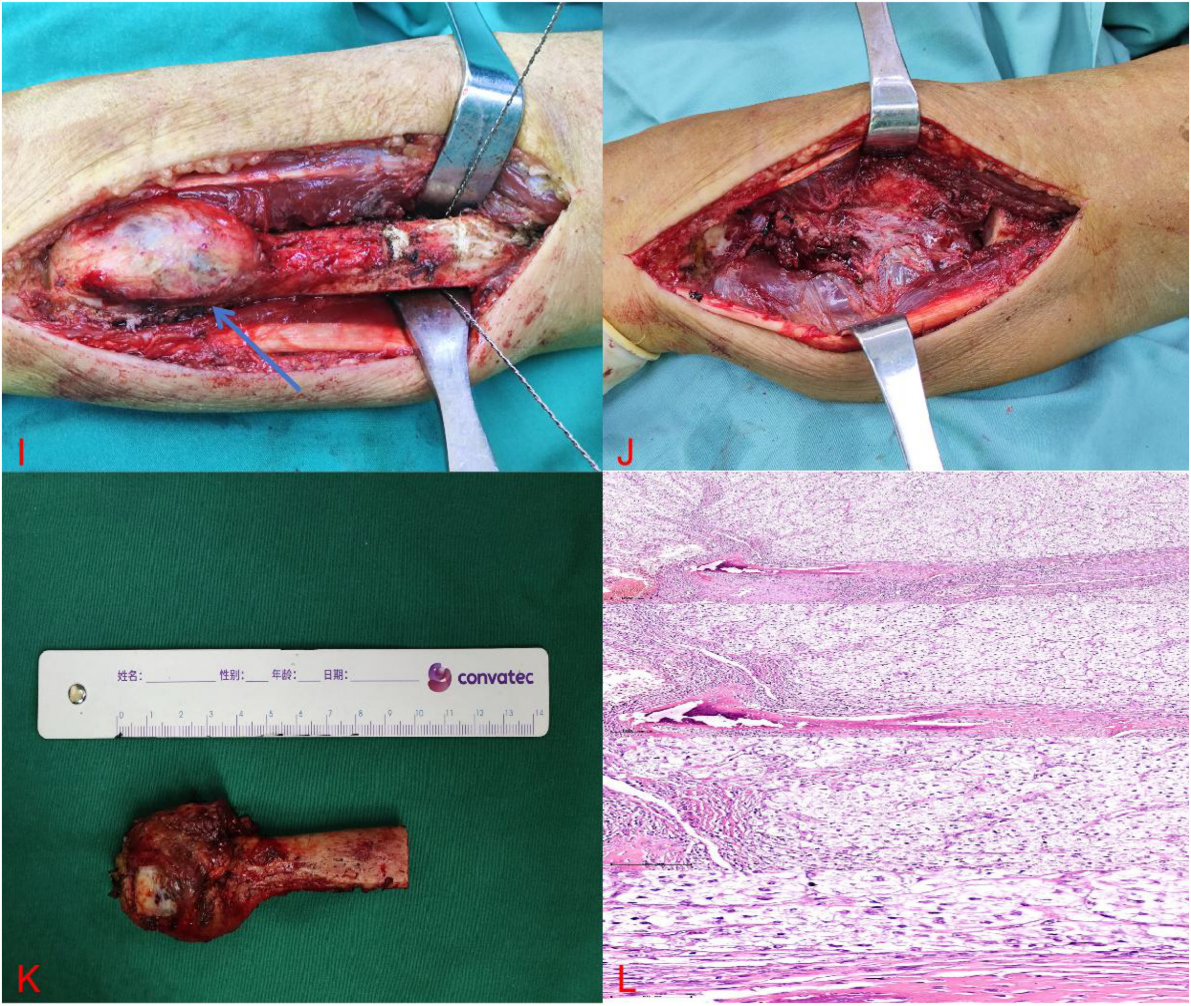
**(I)** Full exposure of the tumor during the operation. **(J)** Enlarged tumor bone after resection. **(K)** surgical field after resection. **(L)** postoperative pathology.

**Figure 3:**
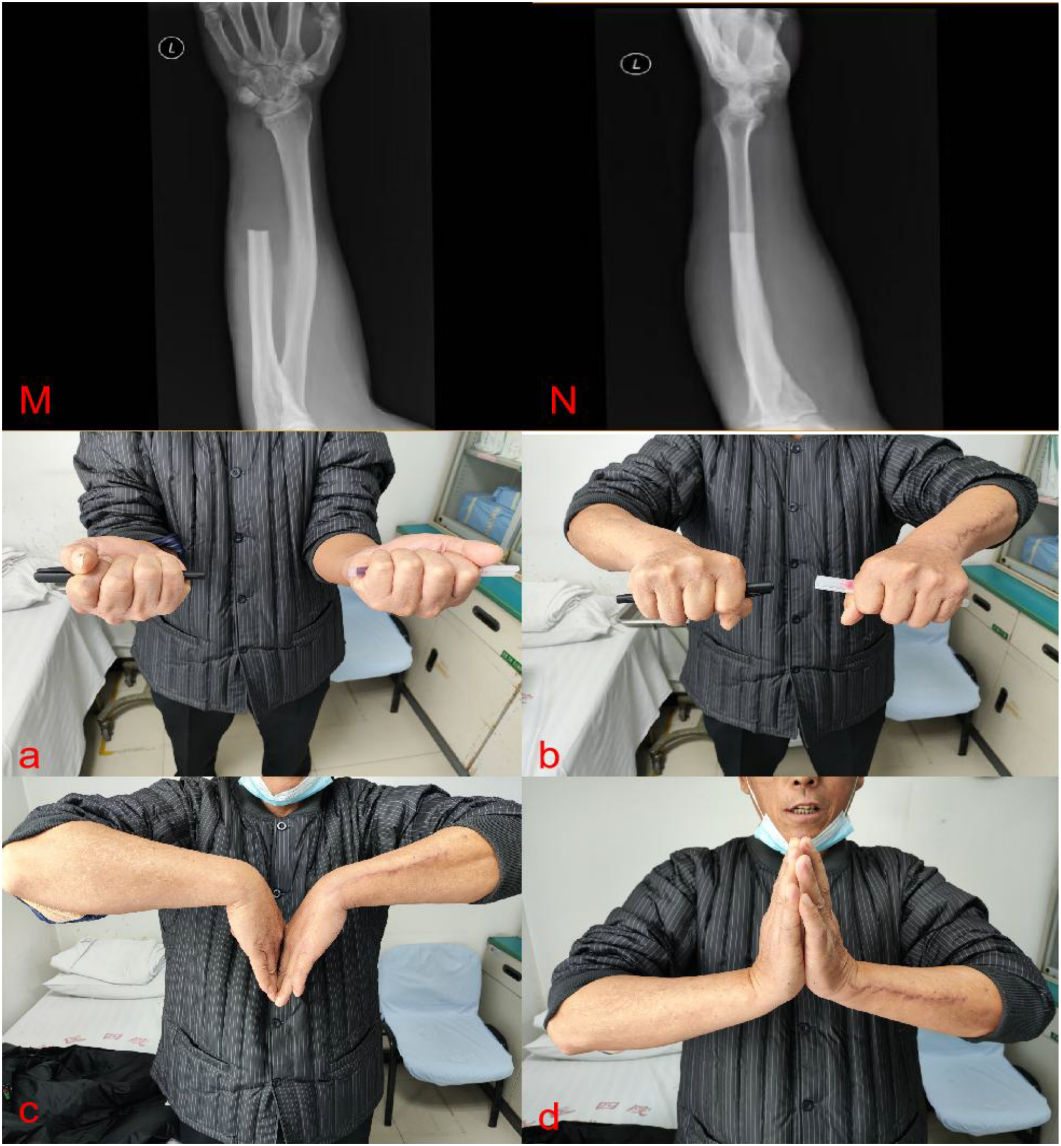
Postoperative radiographs of the ulna and radius. **(M)** positive position. **(N)** lateral view The patient could complete the full ROM, **(a)** pronation. **(b)** supination. **(c)** Palm flexion. **(d)**, dorsiflexion.

A 61-year-old male patient presented to the hospital with persistent pain and swelling in his left forearm for one month. Physical examination reveals significant swelling in the distal part of the left forearm, with obvious local tenderness. The range of motion of the left wrist joint, including pronation and supination, is limited compared to the contralateral side due to pain. X-rays of the left ulna and radius revealed an extracompatimental tumor in the distal ulna (**Figure 1**). Further CT scan showed a destructive bone lesion in the distal ulna (**Figure 1**). MRI images demonstrated a soft tissue mass in the distal ulna, measuring approximately 3.7 cm × 3.6 cm × 3.4 cm (**Figure 1**). Based on imaging analysis showing significant bone destruction and a soft tissue mass in the distal ulna, a preliminary differential diagnosis of bone malignancy was made, including aggressive giant cell tumor, osteosarcoma, chondrosarcoma, and metastatic bone tumor. The patient underwent further head, chest, and abdominal CT scans, which showed no obvious metastatic lesions. Bone scan results indicated abnormal bone imaging agent accumulation in the pelvis and left forearm. We performed a pelvic CT scan and found no obvious bone destruction. We will conduct regular follow-up examinations of the patient’s pelvis. Notably, The patient underwent a laparoscopic radical nephrectomy for left renal clear cell carcinoma in 2023. The tumor was staged as T2N0M0, corresponding to clinical stage II. Following surgery, the patient received regular follow-up examinations and did not undergo postoperative radiotherapy, chemotherapy, or targeted immunotherapy. Given his medical history, bone metastasis from renal cancer was highly suspected. Subsequent lesion biopsy confirmed metastatic clear cell renal cell carcinoma. Preoperative assessments showed a Disabilities of the Arm, Shoulder and Hand (DASH) score of 16, Musculoskeletal Tumor Society (MSTS) score of 23, and Mayo Wrist Score (MWS) of 70. Before the surgery, our department conducted a joint (Multidisciplinary Diagnostic Team) with Oncology and Urology. Due to the patient’s wrist joint pain, limited mobility, and pathological fracture of the distal ulna, finally we performed a wide segmental resection of the distal ulna tumor with a 10 cm resection length. No soft tissue reconstruction was performed—specifically, no ulnar flexor carpi or flexor digitorum superficialis transplantation, nor pronator teres reconstruction—and no implants were used, such as prostheses, bone cement, allograft bone, or autograft bone (**Figure 2**). The postoperative pathological result shows renal clear cell carcinoma with bone metastasis (**Figure 2**). After surgery, the patient underwent follow-up X-rays (**Figure 3**). Considering the patient’s tumor stage is T2N0M1, clinical stage IV, the patient undergoes regular follow-up every three months and receives monthly bone protective agent denosumab for bone metastasis treatment. Postoperative radiotherapy, chemotherapy, and targeted immunotherapy were not applied. Three months postoperatively, the patient’s wrist joint function had recovered to meet daily needs (**Figure 3**). The DASH score at three months postoperatively was 8, MSTS score was 28, and mayo score was 93. The 11 months following the surgery, the DASH score was 7, MSTS score was 29, and mayo score was 94. To date, the patient maintains good tumor control and functional status without any related complications. The patient’s wrist joint range of motion (ROM) has fully recovered, allowing normal joint function.

## Discussion

Primary or metastatic tumors of the distal ulna are very rare. The challenge lies in achieving complete tumor resection while maintaining good wrist joint function. The most common tumor in the distal ulna is giant cell tumor of bone. The surgical treatment options for primary giant cell tumor of bone include curettage and bone grafting, or en bloc resection. However, for malignant tumors metastasizing to the distal ulna, the surgical approach typically involves wide resection of the tumor segment, with soft tissue reconstruction (reattachment of the extensor carpi ulnaris, flexor carpi ulnaris, and pronator quadratus muscles) and implantation of prostheses, bone cement, allografts, or autografts, or even amputation. Although metastasis to the distal ulna is rare, patients with metastatic tumors of the distal ulna may require surgical treatment. As the tumor cells continue to spread, they progressively invade the cortical and medullary bone of the distal ulna. If the tumor involves the wrist joint surface of the distal ulna, it may affect wrist joint function. Moreover, during pronation and supination movements of the forearm, the distal ulna and radius bear significant torsional loads. In this situation, the risk of pathological fractures of the distal ulna increases. Therefore, conservative treatment is rarely recommended for metastatic tumors of the distal ulna due to the increased risk of fractures and joint involvement.

Simple distal ulnar resection (Darrach procedure) or its modified techniques were initially used for degenerative diseases such as distal radioulnar arthritis, but for distal ulnar tumors, a more extensive resection involving greater bone and soft tissue removal is necessary. Additionally, due to the extensive resection required for giant cell tumors or metastatic tumors in the distal ulna, part of the periosteum of the ulna needs to be stripped. After extended distal ulnar resection, the stabilizing effect of the periosteal sleeve of the ulna is lost. Moreover, a large part of the soft tissue capsule is also resected during tumor surgery, which can lead to instability of the distal ulnar stump. This instability may result in postoperative complications such as ulnar deviation of the carpal bones, limited pronation and supination of the forearm, and ulnar stump impingement on the radius, potentially increasing the risk of pain and extensor tendon rupture (4). Distal ulnar resection for giant cell tumor or metastatic tumors is generally performed in young patients who have high demands for wrist joint function, making the issue of wrist joint stability even more critical.

There is still controversy over whether the residual ulnar stump is stable after distal ulnar resection. Some scholars advocate no reconstruction at all, while others support various stabilization methods. In a study of 8 patients with distal ulnae (including 4 cases of giant cell tumor [GCT] of the distal ulna) and various tumors, Cooney et al. (5) treated them only with en bloc resection. Seventy-five percent of the cases achieved good functional and oncological outcomes. They concluded that reconstruction of the distal ulna is not a routine indication. Wolfe et al. (6) believed that after simple resection of the distal ulna, weakness, ulnar-radial convergence, and ulnar translation of the carpal bones may occur; therefore, they advocated reconstruction. Goldner and Hayes (7) initially described the method of stabilizing the ulnar stump after ulnar resection using the ulnar wrist extensor tendon. Subsequently, Kayias et al. (8) first described the application of this technique in giant cell tumors of the distal ulna. At final follow-up, the patients had excellent tumor control and functional outcomes. Badaruddin et al. (9) retrospectively studied 10 patients who underwent distal ulnar resection for GCT between 2014 and 2019; 5 cases underwent extensor carpi ulnaris (ECU) tendon fixation, and 5 cases did not receive ECU tendon fixation. At 12 months, the Mayo Wrist Score (MWS) and Musculoskeletal Tumor Society (MSTS) scores reported by the two groups were similar. Ioannis D et al. (10) analyzed 9 cases of distal ulnar resection for GCT between 2007 and 2015 and concluded that the distal ulna can be widely resected, and satisfactory local disease control and functional outcomes can be achieved regardless of whether the residual ulnar stump is stabilized.

Meanwhile, some authors suggest performing prosthetic replacement after ulnar resection. Nei et al. (11) achieved successful oncological and functional outcomes by performing radical resection of giant cell tumor of the distal ulna, and reconstructing with a constrained total joint arthroplasty. Although prosthetic replacement for tumors in the ulna has shown good short-term results, as Berger (12) pointed out, the lack of soft tissue coverage on the ulna surface due to excessive loss of ulna length hinders its application; it also involves high cost and prolonged operation time.

Some scholars believe that the distal radioulnar joint (DRUJ) and the triangular fibrocartilage complex (TFCC) are important mechanical structures because they can transfer stress to the forearm. Fixing the distal radius with iliac or fibular strut grafts to reconstruct distal ulnar support helps restore the anatomy of the DRUJ and normalizes stress distribution throughout the wrist, thereby maintaining wrist function. Performing ulnar support arthroplasty using an unstable residual iliac bone graft at the end to reconstruct the DRUJ is a relatively simpler method. Naik et al. (13) resected the distal ulna en bloc and transplanted iliac bone fixed to the ulnar side of the radius with screws and Kirschner wires as support. After one year of follow-up, the results were good.

Considering the elderly patient’s age, low postoperative labor capacity requirements, the patient and family’s preference for minimizing surgery time and risks, and financial constraints, we have chosen ulnar head resection after consultation with the family.

## Conclusion

In this case, the patient had complete wrist function at short-term(eleven months) follow-up after simple resection with bone enlargement of the distal ulna tumor, without reconstruction or prosthesis placement. To contextualize this case, **Table 1** presents a summary of various surgical procedures and outcomes for invasive tumors of the distal ulna over the past 20 years. The results show that extensive resection of the distal ulna can achieve good oncological control and functional outcomes. One method to stabilize the ulnar stump is tendon reconstruction. However, according to our study, such reconstruction is optional, and considering the cost and operation time, prosthetic implants and bone grafts do not demonstrate significant benefits. Nevertheless, larger sample sizes and longer-term studies are needed to validate these findings. However, these findings still provide important research data on whether surgical reconstruction or prosthetic implantation is performed for aggressive tumors of the distal ulna, and will promote further developments in the surgical treatment of distal ulna tumors.

**Table 1:** Clinical cases of distal ulna tumors documented in global medical literature over the past two decades.

Surgical methods	Author	Year	Cases	Disease type	Follow-up	Ending
Simple resection	Cooney WP, et al (5)	1997	8 cases Simple resection	4cases GCT 4cases malignant GCT	79 months	Functional results are good. Grip strength was reduced by an average of 15 percent compared to contralateral strength. No need for conventional reconstruction
	Dhinsa BS, et al (14)	2014	12 cases Simple resection	6cases GCT 6cases malignant GCT	64 months	Simple resection can shorten the operation time and the final functional outcome is satisfactory
	Papanastassiou ID, et al (10)	2019	9 cases (4 excision, 5 ECU reconstruction)	GCT	3.6 years	Satisfactory outcome, stability is not a mandatory measure
	Sahito B, et al (9)	2022	10 cases (5 excision, 5 ECU reconstruction)	GCT	1 year	The ECU tendon fixation group (6 months) had good short-term follow-up results, but the mid-term follow-up (12 months) had the same outcome
Soft tissue reconstruction	Singh M, et al (15)	2009	2 cases ECU	GCT	3 years	Good function, no recurrence and metastasis, no evidence of stability > instability
	Mujaddid I, et al (16)	2020	2 cases ECU	GCT	10 months	No recurrence or metastasis; MSTS 25 points MSTS 24 points
	Sharma V, et al (17)	2020	1 cases ECU	GCT	1 year	No recurrence of metastasis, complete function
	Solichin I, et al (18)	2021	1 case ECU + FCU	GCT	6 months	Functional limitations existed within 3 weeks, and full function was restored after 6 months
	Fujihara N, et al (19)	2021	1 cases ECU	GCT	27 months	The tumor returned 22 months later
	Kandarkar MM, et al (20)	2023	1 cases ECU	GCT	6 months	Satisfactory outcome
autogenous bone graft	Naik MA, et al (13)	2013	1 case of iliac bone graft + ECU	GCT	3 years	No metastasis and recurrence; The DRUJ structure could be reconstructed by iliac bone graft. ECU improves stability; eukinesia
	Tsai TC, et al (21)	2019	1 case of iliac bone	GCT	1 year	No metastasis and recurrence; eukinesia
	Verma A, et al (22)	2022	1 case fibula graft+ ECU	GCT	1 year	eukinesia
	Kapoor L, et al (23)	2023	8 cases iliac bone graft	GCT	3 years	Low DASH score; No complications; MSTS rating is good - Excellent
prosthesis implantation	Roidis NT, et al (24)	2007	1 case	GCT	2 years	Normal motor function; There were no complications of prosthesis loosening
	Burke CS, et al (4)	2009	1 case	GCT	9 months	End well
	Jones NF, et al (11)	2020	1 case	GCT	2 years	Flexion 70°, extension 70°, pronation and postrotation function normal; No prosthesis loosening; There was no tumor metastasis or recurrence

## Data Availability

The original contributions presented in the study are included in the article/supplementary material. Further inquiries can be directed to the corresponding author.
